# Cross-Linking Amine-Rich Compounds into High Performing Selective CO_2_ Absorbents

**DOI:** 10.1038/srep07304

**Published:** 2014-12-03

**Authors:** Enrico Andreoli, Eoghan P. Dillon, Laurie Cullum, Lawrence B. Alemany, Andrew R. Barron

**Affiliations:** 1 Department of Chemistry, Rice University, Houston, Texas 77005, USA; 2 Energy Safety Research Institute, College of Engineering, Swansea University, Singleton Park, Swansea SA2 8PP, Wales, UK; 3 Shared Equipment Authority, Rice University, Houston, Texas 77005, USA; 4 Department of Materials Science and Nanoengineering, Rice University, Houston, Texas 77005, USA

## Abstract

Amine-based absorbents play a central role in CO_2_ sequestration and utilization. Amines react selectively with CO_2_, but a drawback is the unproductive weight of solvent or support in the absorbent. Efforts have focused on metal organic frameworks (MOFs) reaching extremely high CO_2_ capacity, but limited selectivity to N_2_ and CH_4_, and decreased uptake at higher temperatures. A desirable system would have selectivity (cf. amine) and high capacity (cf. MOF), but also increased adsorption at higher temperatures. Here, we demonstrate a proof-of-concept where polyethyleneimine (PEI) is converted to a high capacity and highly selective CO_2_ absorbent using buckminsterfullerene (C_60_) as a cross-linker. PEI-C_60_ (CO_2_ absorption of 0.14 g/g at 0.1 bar/90°C) is compared to one of the best MOFs, Mg-MOF-74 (0.06 g/g at 0.1 bar/90°C), and does not absorb any measurable amount of CH_4_ at 50 bar. Thus, PEI-C_60_ can perform better than MOFs in the sweetening of natural gas.

Curbing CO_2_ emissions with effective sequestration is among one of the major contemporary environmental and technological challenges^1^,^2^. Organic amines in solution or tethered to high-surface area supports are commonly used for the absorption of CO_2_^3^,^4^,^5^. Although the amines impart intrinsic selectivity to these systems, a major drawback is that the solvent and support add unproductive weight to the absorbent. The CO_2_ absorption capacity (g CO_2_/g absorbent) is maximized when the amount of solvent and support is minimized. Furthermore, removing anything that is not amine (especially solvent) would reduce the energy demand for regeneration (CO_2_ desorption). It would be ideal to exploit the intrinsic selectivity of amine-bearing materials by using the lowest amount of support to maximize uptake, and avoid the energy demand of solvent heating. Polyethyleneimine (PEI) has a high potential for CO_2_ absorption since it has one amine group every two carbon atoms. However, high molecular weight branched PEIs absorb CO_2_ extremely slowly because of their high viscosity. PEIs have been applied in combination with various supports^6^,^7^,^8^,^9^,^10^, and in particular very high PEI loadings were attained using silica foam and mesoporous capsules as supports^8^,^9^. Alternatively higher PEI loadings (CO_2_ absorption capacities) could be obtained by cross-linking PEI. The mass of cross-linker used to convert the PEI to an effective CO_2_ absorbent could be lower than that of an actual support. The cross-linking of PEI with bi-functional reagents is employed in gene transfection using biodegradable bridges to improve transfection efficacy and reduce citotoxicity^11^,^12^. However, there is no known work in the cross-linking of PEI for enhanced CO_2_ capture. Herein, we show how the loading and CO_2_ absorption performance of PEI can be maximized using C_60_ as PEI cross-linker. In particular, the resulting composite material, PEI-C_60_, has excellent CO_2_ absorption capacity at high temperature and very high selectivity both in the presence of N_2_ and CH_4_.

## Results and discussion

Initially, PEI-C_60_ was synthesized with different molecular weights (600, 1,800, 10,000 and 25,000 Da), showing that the CO_2_ capture performance improved with increasing molecular weight (Supplementary Information). Thus all results presented herein are related to branched PEI 25,000 Da. The preparation of PEI-C_60_ is readily scalable. A brown precipitate of PEI-C_60_ is formed upon mixing solutions of PEI and C_60_ dissolved in chloroform and toluene, respectively, in the presence of NEt_3_. PEI-C_60_ is insoluble in water, ethanol and chloroform. The PEI/C_60_ weight ratio was measured as 73/27 using thermogravimetric analysis, corresponding to a C/N weight ratio of ca. 74/26. This value is comparable to the results from X-ray photoelectron spectroscopy (XPS), 70/30, and elemental analysis, 72/28, which corresponds to about 10 molecules of C_60_ per molecule of PEI (M_w_ = 25,000 Da). Covalent functionalization of C_60_ (rather than physical wrapping) is confirmed by NMR spectroscopy, *vide infra*. Since branched PEI has many primary amines, it is likely that PEI would react with several nanocarbon molecules resulting in a highly interconnected network. The surface area of PEI-C_60_ was measured in the order of about 2.7-2.9 m^2^/g (Supplementary Information).

It should be noted that PEI-C_60_ behaves completely differently to a physical mixture or its components. PEI is a viscous material, while PEI-C_60_ is a non-sticky porous composite capable of absorption. The absorption of CO_2_ on solid C_60_, 0.002 g/g (g CO_2_/g absorbent) at 1 bar^13^, is dramatically lower than that of PEI-C_60_ (0.2 g/g at the same pressure). PEI-C_60_ also shows a greater absorption of CO_2_ than PEI-SWNTs (single walled carbon nanotubes) (0.09 g/g)^14^ in agreement with a higher loading of PEI on C_60_ (75% w/w) compared to SWNTs (50% w/w). Additionally, the PEI:C molar ratio of PEI-C_60_ (1:695) is larger than that of PEI-SWNTs (1:2065) showing that C_60_ can accommodate more PEI molecules than SWNTs despite its much smaller aspect ratio. The hydrophilic PEI segregates from the hydrophobic surface of the SWNT^14^, in contrast C_60_ appears fully internalized in the PEI matrix as inter- and/or intra-molecular cross-linker. The incorporation of hydrophobic centres in the PEI media would possibly force the hydrophilic amine moieties to point toward the surface of the material making the reactions with CO_2_ more effective. PEI/G-silica (PEI-impregnated graphene-porous silica sheets) also exhibited high absorption capacity, 0.19 g/g^15^, due, as we speculate, to a comparable hydrophobic-hydrophilic enhancing effect.

The performance of PEI-C_60_ equates or outperforms those of metal organic frameworks (MOFs) particularly at higher temperatures^16^,^17^. Importantly, the performance of PEI-C_60_ at low pressure and high temperature is better than that of Mg-MOF-74, Mg_2_(1,4-dioxido-2,5-benzenedicarboxylate), which has exceptionally high CO_2_ capacity at very low CO_2_ pressure^18^,^19^. The absorption capacity of PEI-C_60_ is more than twice that of Mg-MOF-74 in single-component CO_2_. Moreover, PEI-C_60_ is extremely selective.

**Figure 1:**
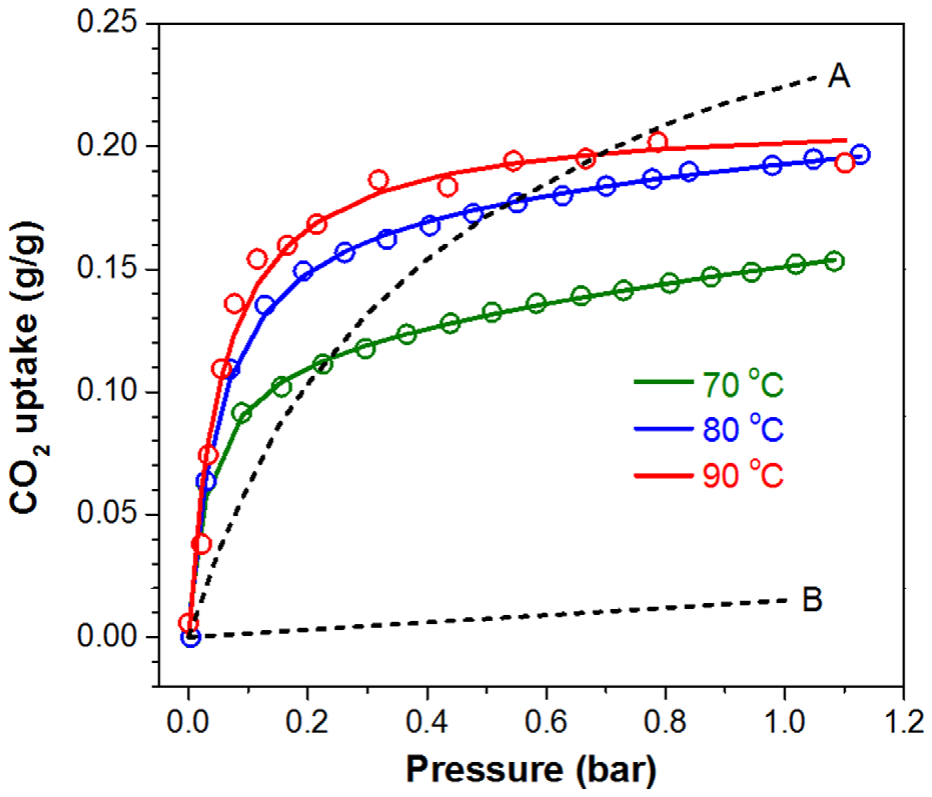
CO_2_ uptake of PEI-C_60_. Comparison of the CO_2_ uptake of PEI-C_60_, Mg-MOF-74, and MOF-177 in the low pressure range. The CO_2_ uptake of PEI-C_60_ was measured at 70, 80 and 90°C. The dashed curves are for the absorption of CO_2_ on (A) Mg-MOF-74 and (B) MOF-177 both at 90°C. PEI-C_60_ outperforms Mg-MOF-74 in the capture of CO_2_ at low pressure: the uptake of PEI-C_60_ is twice as much as that of Mg-MOF-74 at 0.1 bar and 90°C. Furthermore, the CO_2_ absorption capacity of PEI-C_60_ increases with increasing temperature in striking contrast with MOFs which capacity decreases with increasing temperature.

The CO_2_ absorption performance of PEI-C_60_ in the low pressure range (Fig. 1) shows uptake increasing with temperature with a CO_2_ absorption capacity (at 1 bar) going from 0.15 to 0.20 g/g at 70 and 90°C, respectively. This is in dramatic contrast with MOFs whose uptake decreases above room temperature. More significant is the uptake at lower pressures. The CO_2_ uptake of PEI-C_60_ surpasses that of Mg-MOF-74 (Fig. 1, dashed curve A) for pressures below 0.7 bar at 90°C. Mg-MOF-74 is one of the best MOFs for the adsorption of CO_2_ at low pressure and high temperature due to the presence of unsaturated Mg sites that have strong affinity to CO_2_^16^,^19^,^20^. As a comparison, without such binding sites the performance of MOF-177^20^, Zn_4_O(1,3,5-benzenetribenzoate)_2_, is poorer (Fig. 1, dashed curve B). In the case of PEI-C_60_, the amine groups appear to have a higher affinity (reactivity) toward CO_2_ making PEI-C_60_ an excellent material for the capture of CO_2_ at very low pressures (already significant at 0.05 bar) and relatively high temperatures (70–90°C) both ideal properties for the absorption of CO_2_ from flue gases^21^.

**Figure 2:**
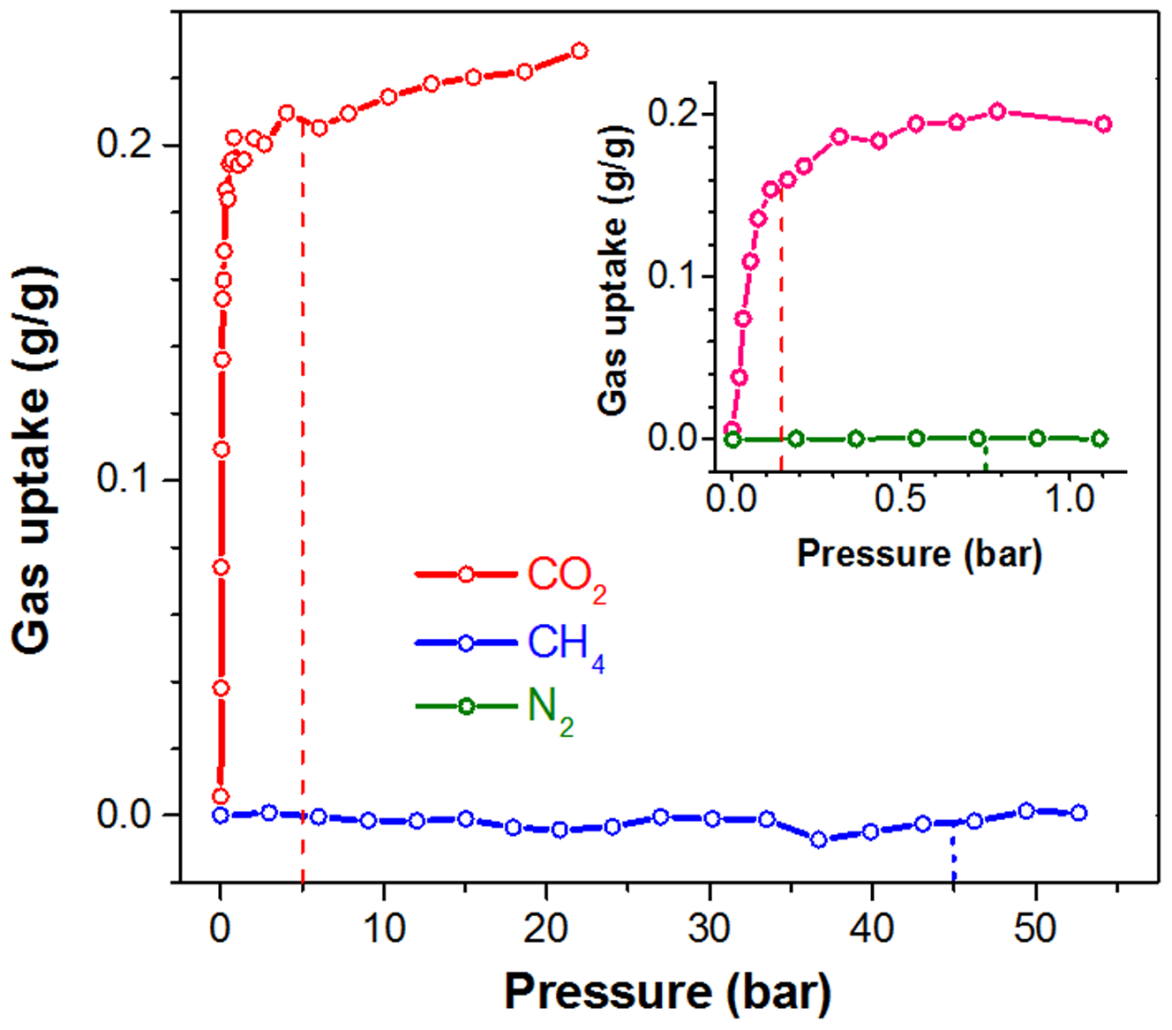
Selectivity of CO_2_ uptake of PEI-C_60_. Single component CO_2_, CH_4_ and N_2_ uptakes of PEI-C_60_ at 90°C in the high pressure range. The dashed lines indicate the corresponding uptakes at the typical pressures of natural gas (5 bar CO_2_ and 45 bar CH_4_) and flue gas (0.15 bar CO_2_ and 0.75 N_2_) in the inset. The outstanding selectivity of PEI-C_60_ is particularly evident for natural gas where PEI-C_60_ reaches full CO_2_ capacity at 5 bar with no significant amount of absorbed CH_4_ at 45 bar.

The absorption performance of PEI-C_60_ in CO_2_, CH_4_ and N_2_ at various pressures is compared in Fig. 2. PEI-C_60_ does not absorb any measurable amount of CH_4_ at pressures up to 50 bar. Practically no N_2_ is absorbed in the range from 0 to 1 bar. Whereas, PEI-C_60_ reaches almost its full CO_2_ absorption capacity, 0.2 g/g, at 1 bar. This very high selectivity has two practical implications. One is related to the capture of CO_2_ from flue gas^21^, assuming that the partial pressures of CO_2_ and N_2_ in the flue gas are 0.15 and 0.75 bar, respectively, PEI-C_60_ would absorb about 0.15 g/g of CO_2_ and 0.0005 g/g N_2_ at 90°C (inset of Fig. 2). This compares to 0.14 g/g of CO_2_ and 0.002 g/g of N_2_ at 25°C for mmen-Mg_2_(dobpcd), mmen = N,N'-dimethylethylenediamine and dobpdc^4−^ = 4,4'-dioxido-3,3'-biphenyldicarboxylate, an amine-functionalized expanded MOF-74 structure^22^. Thus, PEI-C_60_ has promise for capturing CO_2_ from N_2_-rich hot flue gases.

A second major implication is important for natural gas sweetening^23^,^24^. Of particular interest is CO_2_ removal at the wellhead where the gas is typically at >50°C. To date physical adsorbent like activated carbons, zeolites and MOFs have not been able to replace in large-scale amine scrubbing solutions because of their lack of selectivity toward the capture of CO_2_. Selectivity is essential for natural gas sweetening because if the absorbent captures both CO_2_ and CH_4_ an extra step must be added in order to recover that part of final product captured in the absorbent. This is not necessary with PEI-C_60_, the removal of CO_2_ from natural gas at 50 bar, roughly made of 5 bar CO_2_ and 45 bar CH_4_, would give maximum absorption capacity for CO_2_ of 0.2 g/g at 5 bar and no measurable absorption for CH_4_ at 45 bar (Fig. 2). This compares to 0.35 g/g for CO_2_ and 0.1 g/g for CH_4_ at the same pressures and 70°C for Mg-MOF-74, which would require the recovery of large amounts of CH_4_ captured by the absorbent following the CO_2_ removal step^25^.

**Figure 3:**
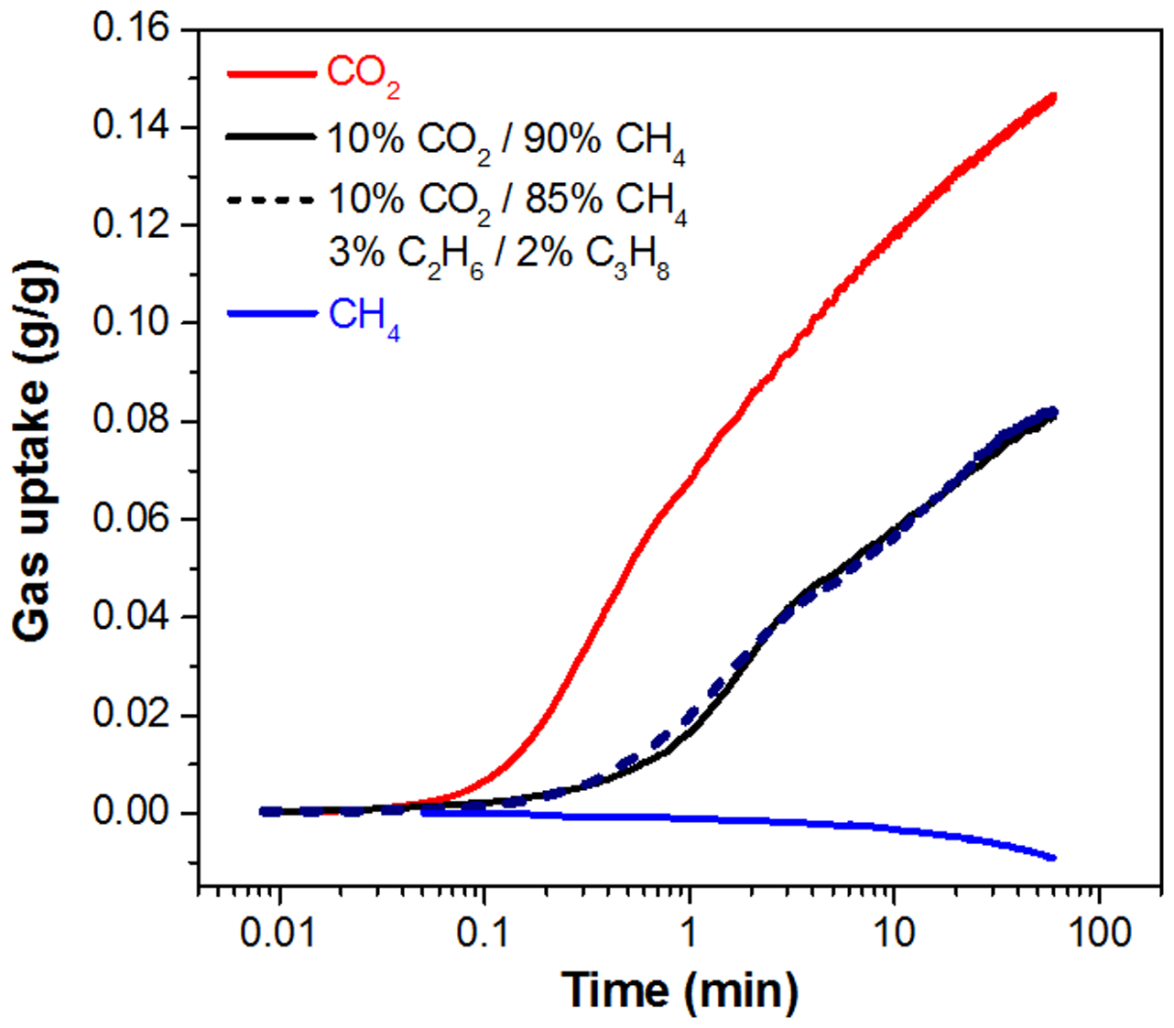
CO_2_ uptake from simulated natural gas mixtures. CO_2_ uptake of PEI-C_60_ from single- and multi-component mixtures of CO_2_, CH_4_, C_2_H_6_, and C_3_H_8_ at 1 atm and 90°C. The decrease of mass in CH_4_ is likely due to a progressive drying of the absorbent. The superposition of the absorption curves in 10% CO_2_/90% CH_4_ and 10% CO_2_/90% CH_4_/3% C_2_H_6_/2% C_3_H_8_ further indicates the lack of interaction of PEI-C_60_ with hydrocarbons.

The absorption of CO_2_ by PEI-C_60_ from mixtures with CH_4_ and simulated natural gas at atmospheric pressure (Fig. 3) is about 0.15 g/g (after 60 min.), while CH_4_ is not absorbed. In the case of the two 10% CO_2_ mixtures, balanced with CH_4_ alone or CH_4_, ethane and propane, the two absorption curves are almost identical. The amount of CO_2_ captured in this case is about 0.08 g/g after 60 min. exposure, more than 50% of what is absorbed in single-component CO_2_, 0.15 g/g. This is a further evidence of the high affinity of PEI-C_60_ toward CO_2_, in fact the capture performance of PEI-C_60_ is five times better than that expected from a simple proportionality between absorption and dilution factor, i.e., 50% of the maximum capacity from a 10% diluted CO_2_.

The performance of PEI-C_60_ was also analysed at atmospheric pressure with thermogravimetric analysis using dry and wet CO_2_. A total uptake of about 0.21 g/g CO_2_ was measured and confirmed with elemental analysis showing that moisture in the feeding gas does not affect the CO_2_ capture performance. Moreover, PEI-C_60_ has a relatively low temperature of regeneration (<90°C) when compared to the amine scrubbing processes (120–130°C), in agreement with what we previously observed with other PEI-modified nanocarbons^14^,^26^. PEI-C_60_ is relatively stable upon cycling maintaining more than 60% of its starting absorption capacity after 100 absorption/desorption cycles at 90°C (Supplementary Information).

**Figure 4:**
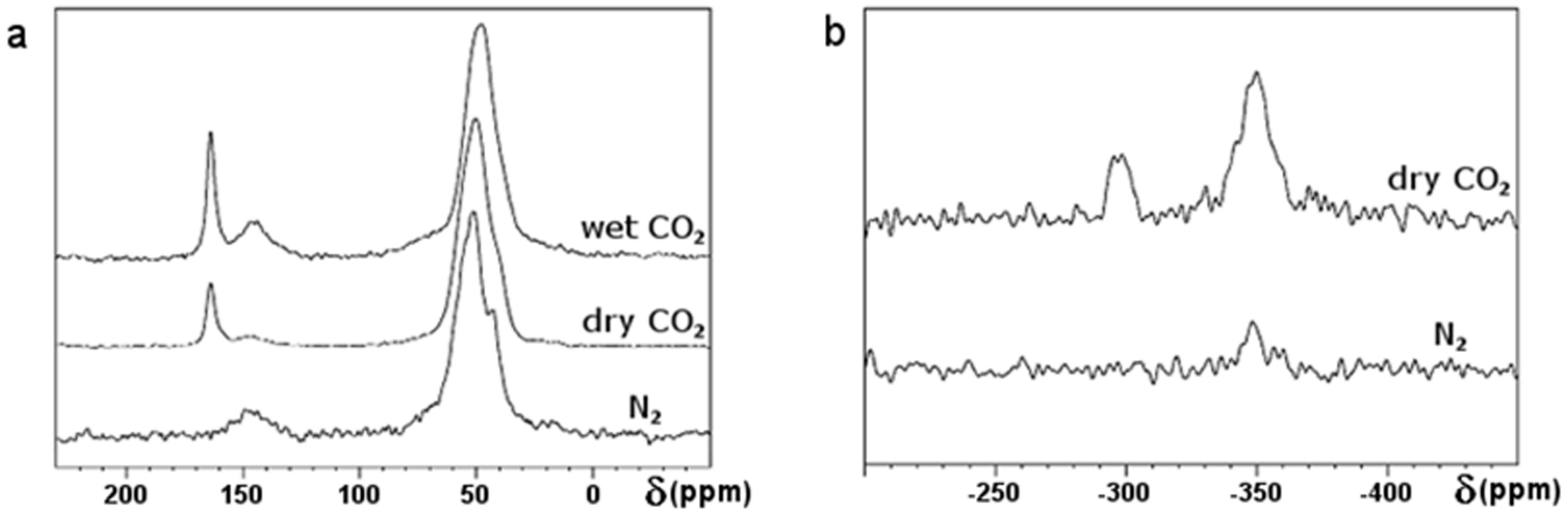
Solid state NMR of PEI-C_60_. Solid state (a) ^1^H-^13^C and (b) ^1^H- ^15^N CP-MAS NMR characterization of PEI-C_60_ after conditioning in wet CO_2_, dry CO_2_ or N_2_.

The chemical species formed upon absorption of CO_2_ in PEI-C_60_ were analysed using nuclear magnetic resonance (NMR). The ^13^C NMR spectra (Fig. 4a) do not allow a definitive differentiation of the carbamate carbonyl signal from the bicarbonate carbonyl signal that may be present. Two bands are present in all ^13^C CP-MAS NMR spectra: one with a peak maximum at 50 ppm (sp^3^ carbons of PEI) and a weaker band with a peak maximum at about 150 ppm (sp^2^ carbons of functionalized C_60_). The former band has a shoulder at about 75 ppm consistent with the presence of sp^3^ nitrogen-substituted carbon atoms on C_60_, as seen for the sidewall functionalization of SWNTs^27^,^28^. A third sharper signal (164 ppm) is also evident in the spectra of samples exposed to wet or dry CO_2_ (but not in the spectrum for wet N_2_). Since this signal can be attributed to carbonate and/or carbamate species, we cannot readily determine the relative contributions of these two species in PEI-C_60_ conditioned in CO_2_. Fortunately, ^15^N CP-MAS NMR presents a much more secure way to determine the presence of carbamate in the presence of bicarbonate. The ^15^N CP-MAS NMR spectra of the PEI-C_60_ conditioned in N_2_ and dry CO_2_ are given in Fig. 4(b). In the ^15^N spectrum recorded after conditioning in dry CO_2_, the band at about -347 ppm can reasonably be assigned to PEI amine nitrogen environments, while the signal at about -297 ppm can reasonably be assigned to PEI-NH-COO^−^ carbamate species. In the sample conditioned in N_2_, the only appreciable signal, after more than 80,000 scans, was that of the PEI amine nitrogens. The XPS characterization of PEI-C_60_ conditioned in wet CO_2_ also supports the formation of bicarbonate and/or carbamate species (Supplementary Information).

With PEI-C_60_, we introduce a new class of materials where specifically selected cross-linkers are used to convert amine-rich compounds into effective CO_2_ absorbents. The C_60_ cross-linker can be depicted as the final result of a progressive shrinkage of a carbon support where PEI increasingly loses contact with the scaffold, as this shrinks, to end suspended between single C_60_ anchoring points. In this way, the amount of support is minimized in order to maximize the amine content and the CO_2_ absorption capacity. This simple approach redefines the way we think about preparing CO_2_ absorbents from anchoring amine compounds to a support to making the amine materials self-supporting with the aid of cross-linkers. We propose that the hydrophobic nature of C_60_ is responsible for the externalization of the hydrophilic amine groups of PEI boosting the absorption performance of the polymer. Accordingly, other cross-linkers could improve this or other critical properties of the resulting composites to achieve further enhanced CO_2_ capture performance with associated reduction in the cost of materials. These new composites could allow for a more efficient capture of CO_2_ and, when integrated in sequestration and utilization technologies, for the containment of the adverse effects of CO_2_ on the environment.

## Methods

### Materials

All materials were used as received. Fullerene C_60_ (99.5%) was purchased from Alpha Aesar, polyethyleneimine branched (PEI, M_w_ = 25,000 Da) and chloroform (≥99.8%) from Sigma Aldrich, toluene (99.98%) from OmniSolv EMD, and triethylamine (99%) from Acros. Ar, N_2_ and CO_2_ high purity gases were all purchased from Matheson TRIGAS. Certified multi-component CO_2_ mixtures were obtained from Applied Gas, Inc.

### Synthesis

PEI-C_60_ was prepared by adding a PEI/chloroform solution (1.00-1.20 g PEI in 35 mL CHCl_3_) to a C_60_/toluene solution (0.12 g C_60_ in 150 ml toluene with 6 mL NEt_3_) while rapidly stirring. A dark-brown PEI-C_60_ precipitate was formed and filtered on a 0.45 μm pore PTFE filter. The precipitate was washed with excess CHCl_3_ and transferred to a clean flask where 50 ml CHCl_3_ was added. The precipitate was bath sonicated for 10 min and again filtered and washed as before. The PEI-C_60_ precipitate was left drying in air overnight and collected as a clustery/rubbery brown solid.

### Equipment

All low and high-pressure gas absorption isotherms were collected with a Setaram PCTPro volumetric apparatus using at least 100 mg of sample. The absorption isotherms at atmospheric pressure were collected with a TA Instrument SDT Q600 thermogravimetric apparatus using at least 5 mg of sample. In this case, the CO_2_ was used either in dry or wet form. Dry CO_2_ was prepared using a stainless still bubbler filled with 3 Å molecular sieves (vacuum dried at 250°C overnight) through which the CO_2_ was passed at room temperature and 50 psig. Wet CO_2_ was prepared using a stainless still bubbler filled with deionized water (bubbled with high-flow CO_2_ at atmospheric pressure and slow-flow CO_2_ at 50 psig for 1 h each) through which the CO_2_ was bubbled at room temperature and 2 psig. This last procedure was also used to prepare wet N_2_. All gases were at ambient pressure when in contact with the absorbent. All uptake values are given in the unit of g/g, i.e., in weight of CO_2_ (in g) per unit weight of absorbent (in g). A Cosctech ECS 4010 Nitrogen/Protein Analyzer was used for CHNO elemental analysis. Linear calibrations obtained with acetanilide standard were used for all elements (R^2^ > 0.999). The solid state ^1^H-^13^C and ^1^H-^15^N CP-MAS NMR spectra were obtained at room temperature using a Bruker AVANCE-III spectrometer (50.3 MHz ^13^C, 20.3 MHz ^15^N, 200.1 MHz ^1^H). Chemical shifts are reported relative to glycine defined as 176.46 ppm for the carbonyl carbon^29^ and -347.58 ppm for the nitrogen^30^. All of the ^13^C spectra were obtained with a 1ms contact time, 32.8ms FID with spinal64 decoupling, 5s relaxation delay, and with 50 Hz (1 ppm) of line broadening applied to the FID. The number of scans varied: 20,512 with PEI-C_60_ conditioned in wet CO_2_ and 16,800 each in the case of N_2_ and dry CO_2_. All of the ^15^N spectra were obtained with a 3ms contact time, 20.5ms FID with spinal64 decoupling, 5s relaxation delay, and with 20 Hz (1 ppm) of line broadening applied to the FID. The number of scans varied: 65,000 for dry CO_2_, 82,000 for N_2_, and 1648 for ammonium carbamate (ammonium signal at -358.1 ppm, carbamate signal at -300.9 ppm). The rotors were packed in a glove bag filled with the same gas used for the conditioning of the absorbent. The XPS spectra were acquired using a Physical Electronics PHI Quantera SXM equipped with an Al X-ray monochromatic source (K_α_ 1486.6 eV at 50.3 W) set at a 200 μm beam diameter and a 45° incident angle. The spectra were collected in ultra-high vacuum conditions (~10^−9^ Torr). The high-resolution spectra were deconvoluted into overlapping peaks using mixed Gaussian-Lorentzian curves after subtraction of a Shirley-type background. The SEM images of the PEI-C_60_ composites were collected using a FEI Quanta 400 ESEM at an accelerating voltage of 10-20 kV and high vacuum (< 5 × 10^−9^ Torr). The surface morphology and area of the PEI-C_60_ composites were characterized using a Veeco Nanoscope IIIA Atomic Force Microscope and a Quantachrome Autosorb-3B Surface Analyzer, respectively.

## Author Contributions

E.A., E.P.D. and L.C. performed the experiments and analysed the data. L.B.A. performed NMR analysis. E.A. created the Figures. E.A. and A.R.B. wrote the manuscript. A.R.B. supervised the project.

## Supplementary Material

Supplementary Information Supplementary data
