# Supplementary material for: Cross-Linking Amine-Rich Compounds into High Performing Selective CO2 Absorbents
Source: Sci Rep. 2014 Dec 3;4:7304. doi: 10.1038/srep07304 (PMC4252903; doi:10.1038/srep07304)
Supplement: Supplementary Information — Supplementary data [file srep07304-s1.pdf]

Supplementary Information for

Cross-Linking Amine-Rich Compounds into High Performing Selective CO_2 Absorbents

Enrico Andreoli, Eoghan P. Dillon, Laurie Cullum, Lawrence B. Alemany, Andrew R. Barron*

Correspondence to: arb@rice.edu

This Supplementary Information includes:

PEI-C_{60} Characterization
CO_2 Absorption/Desorption at Atmospheric Pressure
Effect of the Molecular Weight of PEI
Identification of the Chemical Species Involved in the Absorption of CO_2
Figures S1 to S12
Tables S1 to S3

PEI-C_{60} characterization

Thermogravimetric analysis: The results of the thermogravimetric analysis (TGA) of PEI-C_{60} are given in Figure S1. The absorbent was heated up from room temperature to 800 °C at a rate of 2 °C/min. During the scan the temperature was held at the two fixed values of 90 and 360 °C for 1 h and 12 h, respectively. This was done to allow the full completion of their corresponding processes which are the initial conditioning of the adsorbent in Ar and the decomposition of PEI in air, respectively. The weight decrease with time recorded during the second stage is given in the inset.

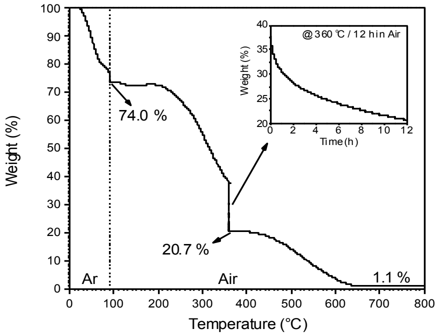

Fig. S1. TGA characterization of PEI-C_{60} heated up from room temperature to 800 °C at a scanning rate of 2 °C/min. The temperature increase was halted and held at 90 °C for 1h and 360 °C for 12 h. The inset shows the weight decrease associated with the second stage.

After conditioning in Ar, the absorbent weight decreased to 74.0% of its initial value. At this point, air was allowed to flow in place of Ar. The weight of the absorbent dropped then to 20.7% after the PEI was decomposed at 360 °C. A further drop to 1.1% was recorded on going from 360 to 650 °C, corresponding to the decomposition of C_{60}. From these results, the portion of PEI is 74.0 – 20.7 = 53.3% and that of C_{60} is 20.7 – 1.1 = 19.6%. The PEI/C_{60} weight ratio can be thus calculated as 53.3/19.6. Of the 53.3 parts of PEI, 29.7 are of C and 17.3 of N, which, together with the C of C_{60}, give an overall C/N weight ratio of about 74/26.

X-ray photoelectron spectroscopy: The X-ray photoelectron spectroscopy (XPS) survey and high resolution spectra of PEI-C_{60} conditioned in N_2 are presented in Figure S2. The two main elements detected are C and N, at about 285 and 400 eV, respectively. A third small signal at about 532 eV is related to the presence of a residual

amount of O. The C/N/O atomic concentration ratio of the three elements is 71.8/27.1/1.1, corresponding to a C/N weight ratio of about 70/30. This value compares reasonably well with that obtained earlier for the bulk material with TGA, 74/26, the difference might be due to the fact that the XPS analysis is confined to a ~ 150 μm^3 volume portion on the surface of the absorbent.

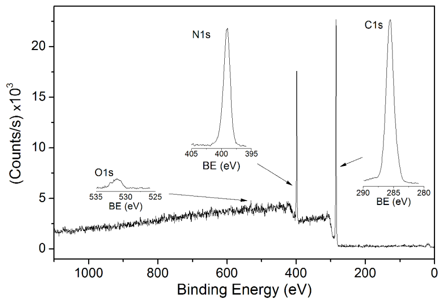

Fig. S2. XPS characterization of PEI-C_{60} after conditioning in N_2. The three peaks C1s, N1s and O1s of the survey spectrum are shown in high resolution in their respective insets.

Surface area measurement: The surface area of PEI-C_{60} was characterized using N_2 adsorbate. In the relative pressure range of 0.05 to 0.30, two portions of the gas adsorption isotherm could be linearized using BET and Langmuir equations, as reported in Figure S3. The surface area of the absorbent is on the order of about 2.7 - 2.9 m^2/g.

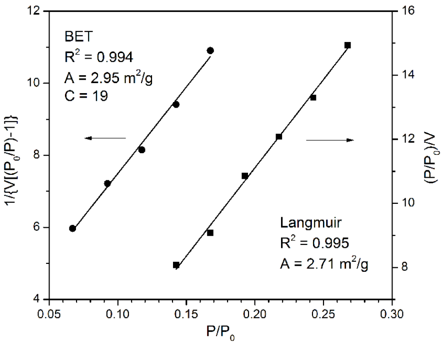

Fig. S3. Linearizations of the N_2 adsorption isotherm of PEI-C_{60}. The two linear fittings correspond to the BET and Langmuir models, respectively.

Scanning electron microscopy: The scanning electron microscopy (SEM) images of as prepared PEI-C_{60} are given in Figure S4. The microporosity of the absorbent is evident at low magnification, Figure S4 (a), where free volume is present between small droplets and larger clots. The porosity is heterogeneously distributed with some regions characterized by pores smaller than a micrometer and others completely compact, as shown in the two images collected at higher magnification, Figures S4 (b) and (c). A close up of one of these last regions, Figure S4 (d), shows that at a scale below 1 μm the material is completely compact excluding the presence of mesopores.

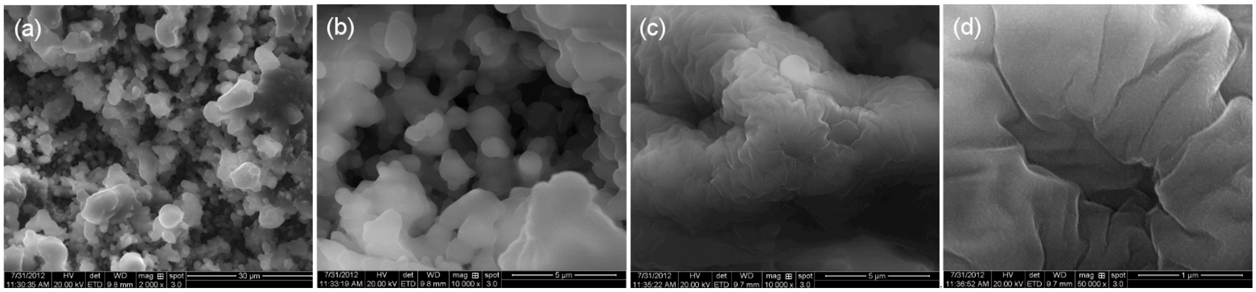

Fig. S4. SEM images of as prepared PEI-C_{60} showing the presence of non-homogeneously distributed microporosity (a, b and c). At the nanoscale level (d) the absorbent appears compact.

Atomic force microscopy: The results of the atomic force microscopy (AFM) characterization of PEI-C_{60} are shown in Figure S5. In this case the absorbent was probe sonicated in water and drop cast on a cleaved mica substrate as a suspension, due to the insolubility of PEI-C_{60} in solvents. As can be seen, the core of a large aggregate in the center of the image is composed of several small particles that are 1 nm in height, indicating that the C_{60} has been internalized and the PEI externalized in a micellar-like structure. The PEI spreads out on the surface due to the hydrophilicity of mica.

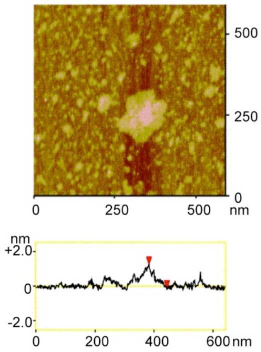

Fig. S5. AFM image and sectional analysis of PEI-C_{60} showing internalized C_{60} and indicating a height for the central cluster of about 2 nm.

Further evidence for the externalization of PEI comes from the SEM images of PEI-C_{60} in Figure S4. The SEM images reveal a highly interconnected network of polymer nodules and given that we used branched PEI with many primary amines, one can expect that one PEI chain would react with several nanocarbon molecules resulting in a highly interconnected network.

CO_2 absorption/desorption at atmospheric pressure

Thermogravimetric analysis: The CO_2 absorption performance of PEI-C_{60} at atmospheric pressure is shown in Figure S6. In this plot there are three gravimetric curves (lower) associated with the same temperature program (upper) used for each of the gravimetric curves. The gases used during the measurements are also indicated above the plots.

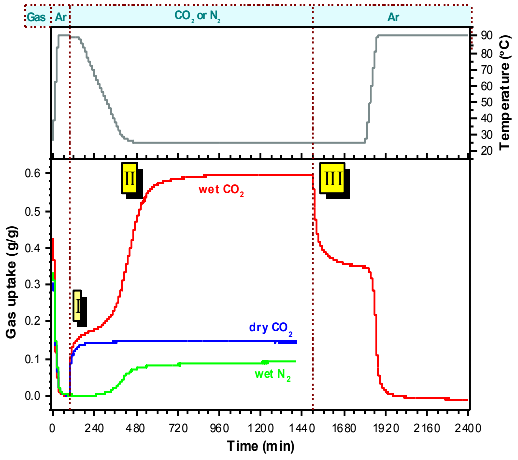

Fig. S6. Thermogravimetric characterization of the CO_2 capture performance of PEI-C_{60}. Initially, the weight of the absorbent falls with time while the temperature is increased in Ar. Then, the weight increases on exposure to wet or dry CO_2, or wet N_2. The absorption of CO_2 as itself, stage I, or with H_2O, as the temperature is decreased, stage II, is followed by a last stage, III, relative to the regeneration of the absorbent in Ar.

Three absorption conditions were investigated: wet CO_2 (red line), dry CO_2 (blue line), and wet N_2 (green line). Wet or dry gases were prepared at room temperature using bubblers filled either with deionized water or dry molecular sieves, respectively. Freshly prepared samples lost about 0.3-0.4 g/g of their weight upon heating to 90 °C in Ar due to the release of moisture and adsorbed gases, *i.e.*, CO_2. The carrier gas was then switched to either wet or dry CO_2 or wet N_2 and the weight increases determined while the temperature was lowered (Figure S6). Both wet and dry

CO_2 showed immediate absorption of 0.16 g/g and 0.14 g/g at 90 °C for wet and dry CO_2, respectively. In contrast, the sample exposed to wet N_2 showed no appreciable mass change until the temperature decreased below 75 °C, at which point a maximum weight increase of 0.09 g/g was observed once the sample reached room temperature. The dry CO_2 sample shows no additional absorption with lowering of the temperature, but the wet CO_2 sample shows a second increase below 75 °C to a maximum of about 0.60 g/g. It is easy to recognize, therefore, that the absorption of CO_2 went through two stages, I and II, in Figure S6. During stage I (between 90 and 75 °C) CO_2 is captured mainly in dry form (*ca.* 0.14 g/g), although a smaller portion associated with H_2O (*ca.* 0.02 g/g) is observed. In stage II (75 °C to room temperature) CO_2 is captured in combination with H_2O and the total amount collectively absorbed during stage II can be calculated from the difference between wet and dry CO_2, *i.e.*, 0.60 - 0.14 = 0.46 g/g. If we consider a H_2O uptake of 0.09 g/g, as from the wet N_2 absorption, the amount of CO_2 absorbed during stage II would be 0.46 - 0.09 = 0.37 g/g. However, this value may be incorrect since the amount of H_2O absorbed with N_2 or CO_2 could be different. This is evident in stage I where no H_2O is absorbed with wet N_2 while it is with wet CO_2. A rationale for this difference could be that the driving force of H_2O absorption for bicarbonate formation is greater than that of amine protonation. The exact amounts of absorbed CO_2 and H_2O can be obtained from the elemental analysis of PEI-C_{60} conditioned in dry Ar and wet CO_2, as reported in Section 2.2.

The regenerative performance of PEI-C_{60} is also presented in Figure S6. The portion of the wet CO_2 curve labeled as stage III shows that upon switching CO_2 to Ar the sample lost at room temperature 40% of the captured mass. Another 60% was lost below 90 °C. Furthermore, PEI-C_{60} maintained more than 60% of its starting absorption capacity after 100 absorption/desorption cycles at 90 °C, as shown in Figure S7.

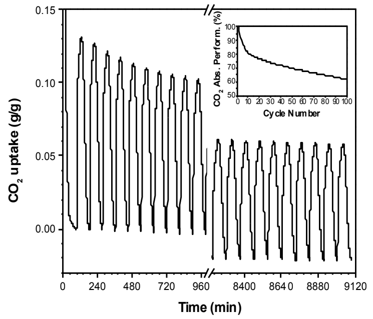

Fig. S7. Thermogravimetric characterization of the CO_2 absorption/desorption performance of PEI-C_{60} at 90 °C. The first and last ten of a total of 100 cycles are presented. In the inset, the CO_2 absorption performance of the absorbent is shown as a function of the number of cycles.

Elemental analysis: The total amounts of absorbed CO_2 and H_2O can be calculated from the comparison of the elemental analyses of PEI-C_{60} conditioned in dry Ar and wet CO_2. The elemental analyses indicate that the chemical formula of PEI-C_{60} conditioned in dry Ar is $CH_{1.73}N_{0.33}$, while that in wet CO_2 is $CH_{2.20}N_{0.31}O_{0.48}$. The stoichiometric balance for the molecular components $(C_2H_5N)_x(C_{60})_y(CO_2)_z(H_2O)_w$ can be then calculated as x = 0.33 and y = 0.0053 (z = w = 0), in dry Ar, and as x = 0.31, y = 0.0050, z = 0.08 and w = 0.32, in wet CO_2. Details of the experimental results and calculations are given as follows.

The PEI-C_{60} samples were conditioned in two environments: 1) dry Ar and 2) wet CO_2. The obtained conditioned samples were kept in containers filled with the same gas used for the conditioning and analyzed. The time per analysis was about 40 min. Three measurements in sequence were performed per sample.

The thermogravimetric profiles for the conditioning of PEI-C_{60} in dry Ar and wet CO_2 are reported in Figure S8.

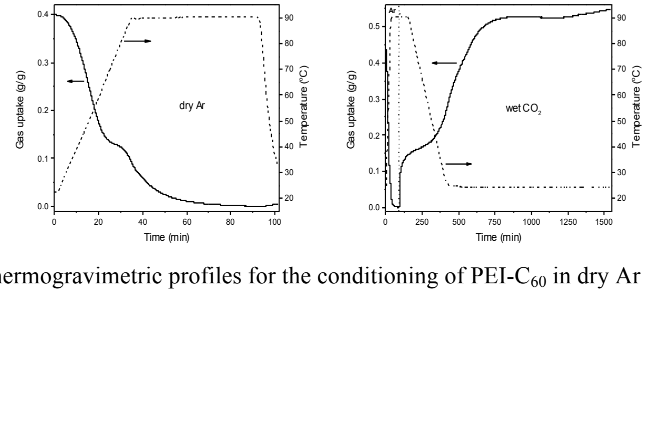

Fig. S8. Thermogravimetric profiles for the conditioning of PEI-C_{60} in dry Ar or wet CO_2.

The sample in dry Ar lost about 40% of its weight at the end of the conditioning, while that in wet CO_2 captured an overall amount of CO_2+H_2O of 0.55 g/g.

The calibration lines for carbon, nitrogen and hydrogen obtained with acetanilide standard are presented in Figure S9.

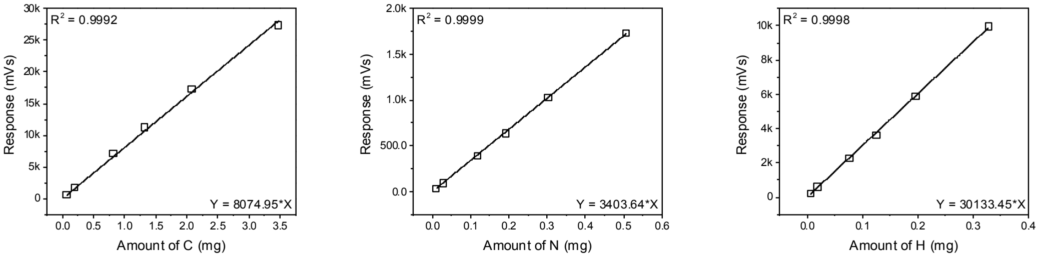

Fig. S9. Calibration lines for carbon, nitrogen and hydrogen (R^2 > 0.999).

The responses for the elemental analysis of PEI-C_{60} are given in Table S1.

Table S1. Responses for the elemental analyses of PEI-C_{60} conditioned in dry Ar and wet CO_2.

	**dry Ar**			**wet CO_2**		
	**Sample 1**	**Sample 2**	**Sample 3**	**Sample 1**	**Sample 2**	**Sample 3**
**Sample weight (mg)**	0.962	1.138	2.982	2.607	0.880	0.782
**Element**	**Response (mVs)**	**Response (mVs)**	**Response (mVs)**	**Response (mVs)**	**Response (mVs)**	**Response (mVs)**
**C**	5060.603	5702.331	14073.066	9650.676	3557.855	3324.929
**N**	813.239	927.124	2427.077	1471.699	498.662	471.47
**H**	2734.697	3293.652	8895.039	6660.998	2076.583	1887.182

These responses are converted to weights using the calibration lines. The weights are then converted into moles from which the stoichiometric ratios can be calculated as presented in Tables S2 and S3.

From Table S2, the N/C stoichiometric ratio is reproducible among all samples, about 0.33. The amount of H increases with time, most likely because of absorption of moisture since the samples were run in chronological order. For the same reason the difference between weighted and calculated weight (Diff%) also increases due to the oxygen of moisture.

Table S2. Results from the elemental analysis of C_{60}-PEI conditioned in dry Ar.

**PEI-C_{60} dry Ar**				
Sample 1	0.962 mg			
Element	Response (mVs)	Amount (mg)	Moles (mol)	Stoich. Ratio
C	5060.603	0.627	5.22E-05	1.00
H	2734.697	0.091	9.00E-05	1.73
N	813.239	0.239	1.71E-05	0.33
TOT		0.956 mg		
Diff%		0.58 %		
Sample 2	1.138 mg			
Element	Response (mVs)	Amount (mg)	Moles (mol)	Stoich. Ratio
C	5702.331	0.706	5.88E-05	1.00
H	3293.652	0.109	1.08E-04	1.84
N	927.124	0.272	1.94E-05	0.33
TOT		1.088 mg		
Diff%		4.41 %		
Sample 3	2.982 mg			
Element	Response (mVs)	Amount (mg)	Moles (mol)	Stoich. Ratio
C	14073.066	1.743	1.45E-04	1.00
H	8895.039	0.295	2.93E-04	2.02
N	2427.077	0.713	5.09E-05	0.35
TOT		2.751 mg		
Diff%		7.74 %		

The sample most representative of PEI-C_{60} conditioned in dry Ar is the first less exposed to the air. The formula obtained for PEI-C_{60} conditioned in dry Ar is then $C_1H_{1.73}N_{0.33}$. From this, it is possible to find the PEI/C_{60} weight ratio as follows:

PEI = $(\text{aziridine})_X = (C_2H_5N)_X$

PEI-$C_{60} = (C_2H_5N)_X\,(C_{60})_Y$

$C_1H_{1.73}N_{0.33} = (C_2H_5N)_X\,(C_{60})_Y = C_{2X+60Y}\,H_{5X}\,N_X$

$1 = 2X + 60Y$; $1.73 = 5X$; $0.33 = X \Rightarrow X = 0.33$, $Y = 0.0053$

$MW(C_2H_5N) = 43.07$ g/mol $\Rightarrow$ PEI = $MW(C_2H_5N)*0.33 = 14.21$ g

$MW(C_{60}) = 720.64$ g/mol $\Rightarrow$ $C_{60} = MW(C_{60})*0.0053 = 3.82$ g

PEI/C_{60} weight ratio = 14.21 / 3.82 = 78.8 / 21.2 .

From Table S3, the N/C stoichiometric ratio is reproducible among all samples, about 0.30. The amount of H decreases with time, most likely because of desorption of captured H_2O since the samples were run in chronological order. For the same reason the difference between weighted and calculated weight (Diff%) also decreases due to loss of captured gases. Note that these differences are due to the presence of oxygen. The amount of oxygen can be estimated as the difference between amount of sample taken and total amount of CHN measured.

Table S3. Results from the elemental analysis of PEI-C_{60} conditioned in wet CO_2.

**PEI-C_{60} wet CO_2**				
Sample 1	2.607 mg			
Element	Response (mVs)	Amount (mg)	Moles (mol)	Stoich. Ratio
C	9650.676	1.195	9.95E-05	1.00
H	6660.998	0.221	2.19E-04	2.20
N	1471.699	0.432	3.09E-05	0.31
O		2.607 – 1.849 = 0.758	4.74E-05	0.48
TOT		1.849 mg		
Diff%		29.09 %		
Sample 2	0.880 mg			
Element	Response (mVs)	Amount (mg)	Moles (mol)	Stoich. Ratio
C	3557.855	0.441	3.67E-05	1.00
H	2076.583	0.069	6.84E-05	1.86
N	498.662	0.147	1.05E-05	0.29
O		0.880 – 0.656 = 0.224	1.40E-05	0.38
TOT		0.656	mg	
Diff%		25.45 %	%	
Sample 3	0.782 mg			
Element	Response (mVs)	Amount (mg)	Moles (mol)	Stoich. Ratio
C	3324.929	0.412	3.43E-05	1.00
H	1887.182	0.063	6.21E-05	1.81
N	471.470	0.139	9.89E-06	0.29
O		0.782 – 0.613 = 0.169	1.06E-05	0.31
TOT		0.613 mg		
Diff%		21.62 %		

The sample most representative of PEI-C_{60} conditioned in wet CO_2 is the first less exposed to the air. The formula obtained for C_{60}-PEI conditioned in wet CO_2 is then $C_1H_{2.20}N_{0.31}O_{0.48}$. From this, it is possible to find the absorbed amounts of CO_2 and H_2O as follows:

PEI = $(\text{aziridine})_X = (C_2H_5N)_X$

PEI-$C_{60} + CO_2 + H_2O = (C_2H_5N)_X\,(C_{60})_Y(CO_2)_Z(H_2O)_W$

$C_1H_{2.20}N_{0.31}O_{0.48} = (C_2H_5N)_X\,(C_{60})_Y(CO_2)_Z(H_2O)_W = C_{2X+60Y+Z}\,H_{5X+2W}\,N_X\,O_{2Z+W}$

$1 = 2X + 60Y + Z$; $2.20 = 5X + 2W$; $0.31 = X$; $0.48 = 2Z + W \Rightarrow$

$\Rightarrow X = 0.31$, $W = (2.20-5*0.31)/2 = 0.32$, $Z = (0.48-0.32)/2 = 0.08$, $Y = (1-2*0.31-0.08)/60 = 0.0050$

$\Rightarrow X = 0.31$, $Y = 0.005$, $Z = 0.08$, $W = 0.32$

$MW(C_2H_5N) = 43.07$ g/mol $\Rightarrow$ $PEI = MW(C_2H_5N)*0.31 = 13.35$ g

$MW(C_{60}) = 720.64$ g/mol $\Rightarrow$ $C_{60} = MW(C_{60})*0.0050 = 3.60$ g

$MW(CO_2) = 44.01$ g/mol $\Rightarrow$ $CO_2 = MW(CO_2)*0.08 = 3.52$ g

$MW(H_2O) = 18.01$ g/mol $\Rightarrow$ $H_2O = MW(H_2O)*0.32 = 5.76$ g

PEI/C_{60} weight ratio = 13.35 / 3.60 = 78.8 / 21.2 .

The PEI/C_{60} weight ratio is exactly as that previously calculated for PEI-C_{60} conditioned in dry Ar. The CO_2 and H_2O uptakes can be calculated as follows:

Captured CO_2 = 3.52 / (13.35+3.60) = 0.21 g/g
Captured H_2O = 5.76 / (13.35+3.60) = 0.34 g/g

The total captured mass is 0.55 g/g, the same as the amount measured with the TGA (Figure S8).

The overall CO_2:H_2O stoichiometric ratio is 0.08:0.32 = 1:4 (as that for the CO_2:N ratio).

The N:H_2O (aziridine:H_2O) stoichiometric ratio is 0.31:0.32 = 1:1.

The total CO_2 capture capacity of C_{60}-PEI is then 0.21 g/g corresponding to 4.8 mmol/g.

It is interesting to note that PEI-C_{60} absorbs mainly H_2O during stage II (Figure S6), but this does not affect the total amount of CO_2 captured in the presence of moisture in the gas feed. Also, the overall CO_2:H_2O molar ratio of 1:4 and a N:H_2O one of 1:1. It seems that PEI-C_{60} absorbs H_2O until each amine group in the absorbent is saturated with one molecule of H_2O.

Effect of the molecular weight of PEI

We have previously shown with PEI-SWNT conjugates that the higher the PEI molecular weight the greater the uptake of CO_2 (*19*). Figure S10 shows the thermogravimetric curves for the absorption of wet CO_2 of PEI-C_{60} prepared with different PEIs, but using the same synthetic procedure and PEI/C_{60} weight ratio. It is clear that the overall absorption of CO_2+H_2O improves with increasing molecular weight. It is important to note that in the case of PEI(600)-C_{60} the adduct melts at the 90 °C. The best absorption performance is obtained with branched PEI 25,000 Da reaching 0.15 g/g uptake in 60 min. at 90 °C and a total of CO_2+H_2O of 0.45 g/g going to 25 °C in other 120 min.

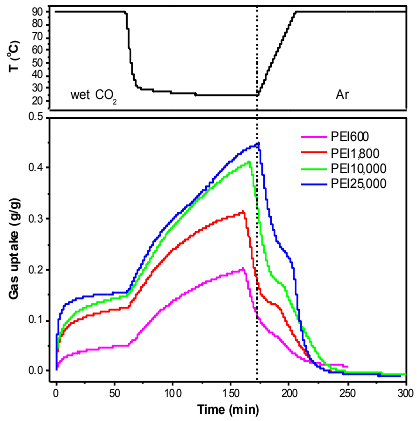

Fig. S10. Thermogravimetric characterization of the CO_2 absorption/desorption performance of PEI-C_{60} at 90 °C prepared from PEIs of different molecular weights.

Identification of the chemical species involved in the absorption of CO_2

Nuclear magnetic resonance: The solid state ^{13}C and ^{15}N NMR spectra of the PEI-C_{60} conditioned in wet CO_2, dry CO_2, and wet N_2 are shown in Figure S11 (note that this figure is the same as Figure 4 of the main manuscript and it is reported here again for convenience). The ^{15}N spectra demonstrate the presence of carbamate. The ^{13}C spectra do not allow a secure differentiation of the carbamate carbonyl signal from the bicarbonate carbonyl signal that may also be present, as will now be discussed.

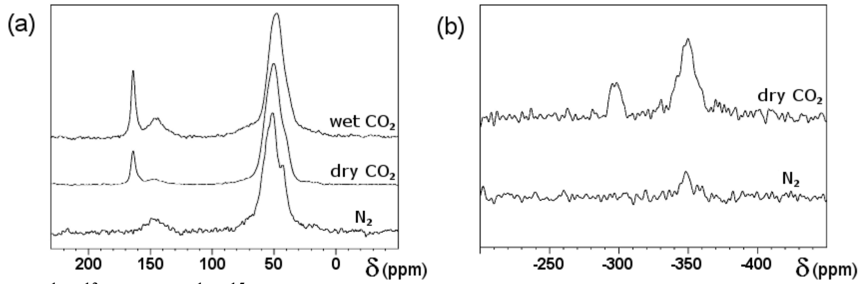

Fig. S11. Solid state (a) ^1H-^{13}C and (b) ^1H-^{15}N CP-MAS NMR characterization of PEI-C_{60} after conditioning in wet CO_2, dry CO_2 or N_2.

Two distinctive bands are present in all ^{13}C CP-MAS NMR spectra, Figure S11 (a): a band with a peak maximum at about 50 ppm from the sp^3 carbons of PEI and a weaker band with a peak maximum at about 150 ppm from the sp^2 carbons of functionalized C_{60}. A third sharper signal at 164 ppm is also evident in spectra for samples exposed to wet or dry CO_2 (but not in the spectrum exposed to wet N_2). Since this signal can be attributed to carbonate and/or carbamate species, we cannot readily determine the relative contributions of these two species in a MAS ^{13}C NMR spectrum of PEI-C_{60} conditioned in CO_2; this is not surprising in light of prior work.

Ammonium bicarbonate gives a single, very sharp MAS ^{13}C NMR signal at 163.5 ppm in agreement with a previous report [1]; the carbonyl peak position of bicarbonate is commonly observed at about 162 ppm in CO_2-amine-H_2O solutions [2]. In contrast, ammonium carbamate ($H_2NCO_2NH_4$) gives a pair of ^{13}C signals differing in peak height and linewidth in an MAS NMR spectrum obtained at a low magnetic field strength [1] because the ^{14}N quadrupole prevents complete averaging of the ^{13}C-^{14}N dipolar coupling [3-5]. The effect becomes greater as the field strength decreases, and the separation of the two signals is field dependent, as demonstrated by the carbonyl lineshape of ammonium carbamate at 25 MHz ^{13}C [1] and at 50 MHz ^{13}C (our work). These aspects significantly complicate analyzing the carbonyl region in an MAS spectrum if both bicarbonate and carbamate are present, as the bicarbonate signal may overlap part of the complex carbamate signal [1] since their chemical shifts are very similar (an aqueous solution of ammonium carbamate gives a signal at 165.9-166.2 ppm [6-7], while solutions of anionic N-alkyl carbamates give a signal at 163-165 ppm [8-12]).

Two other factors cause additional difficulties in interpreting the signal at 164 ppm in the MAS ^{13}C NMR spectrum of PEI-C_{60} conditioned with CO_2. First, the distinctive, very sharp signal of NH_4HCO_3 is significantly broadened when mixed with $H_2NCO_2NH_4$ [1] and thus is no longer as easily recognizable; the same effect would be expected if bicarbonate is present in our sample. Second, the carbamate carbonyl signal may be just a single peak, even at 50 MHz ^{13}C, as shown by the MAS ^{13}C NMR spectrum of CO_2 adsorbed on PEI immobilized on a mesoporous silica support [13]. The half-height linewidth of this signal [13] is similar to that of the carbonyl signals in Figure S11 (a) with the same field strength and same line broadening used.

Fortunately, CP-MAS ^{15}N NMR presents a much more secure way to determine the presence of carbamate in the presence of bicarbonate, and, so, the ^{15}N CP-MAS NMR spectra of the PEI-C_{60} conditioned in N_2 and dry CO_2 were also collected, Figure S11 (b). In the ^{15}N spectrum recorded after conditioning in dry CO_2, the band at about -347 ppm can reasonably be assigned to PEI amine nitrogen environments, while the signal at about -297 ppm can reasonably be assigned to PEI-NH-COO^- carbamate species. In particular, the carbamate assignment is consistent with the ^{15}N chemical shifts reported for a CO_2-treated, amine-modified, nanoporous clay [14] and with the ^{15}N chemical shifts of ammonium carbamate and other simple carbamates [15-18]. In the sample conditioned in N_2, the only appreciable signal, after more than 80,000 scans, was that of the PEI amine nitrogens.

X-ray photoelectron spectroscopy: The XPS characterization of PEI-C_{60} conditioned in wet CO_2 (Figure S12) also supports the formation of bicarbonate and/or carbamate species. The C1s high resolution spectrum shows a peak at 284.6 eV consistent with the presence of the sp^2 C=C bonds of C_{60}, while the two peaks at 285.5 and 286.2 eV are related to the sp^3 C-C and C-N bonds in PEI. A fourth smaller peak is also present at 288.1 eV in agreement with the formation of bicarbonate and/or carbamate species both found in this binding energy region [19-20]. The intensity of this peak cannot be related to the effective amount of CO_2 captured by PEI-C_{60} because most of the CO_2 is likely desorbed in the UHV chamber. The contributions at 531.1 and 532.2 eV in the O1s spectrum are consistent with the presence of two different CO_2 absorption species such as those of bicarbonate and carbamate. The other peak at 533.3 eV is due to a residual trace of H_2O present in the absorbent.

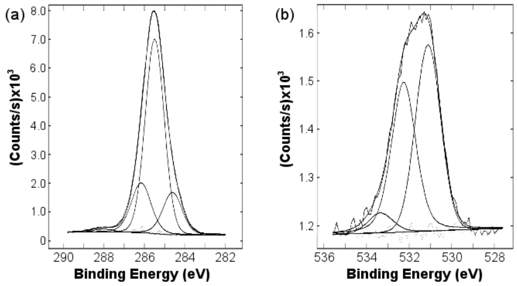

Fig. S12. High resolution (a) C1s and (b) O1s XPS characterization of PEI-C_{60} after conditioning in wet CO_2.

References

1. X. Li, E. Hagaman, C. Tsouris, J. W. Lee, *Energy Fuels* **17**, 69-74 (2003).
2. J. Y. Park, S. J. Yoon, H. Lee, *Environ. Sci. Technol.* **37**, 1670-1675 (2003).
3. J. G. Hexem, M. H. Frey, S. J. Opella, *J. Am. Chem. Soc.* **103**, 224-226 (1981).
4. A. Naito, S. Ganapathy, C. A. McDowell, *J. Chem. Phys.* **74**, 5393-5397 (1981).
5. E. M. Menger, W. S. Veeman, *J. Magn. Reson.* **46**, 257-268 (1982).
6. N. Wen, M. H. Brooker, *J. Phys. Chem.* **99**, 359-368 (1995).
7. F. Mani, M. Peruzzini,; P. Stoppioni, *Green Chem.* **8**, 995-1000 (2006).
8. R. J. Hook, *Ind. Eng. Chem. Res.* **36**, 1779-1790 (1997).
9. A. Dibenedetto, M. Aresta, C. Fragale, M. Narracci, *Green. Chem.* **4**, 439-443 (2002).
10. W. Böttinger, M. Maiwald, H. Hasse, *Fluid Phase Equilib.* **263**, 131-143 (2008).
11. F. Barzagli, F. Mani, M. Peruzzini, *Energy Environ. Sci.* **2**, 322-330 (2009).
12. G-j. Fan, A. G. H. Wee, R. Idem, P. Tontiwachwuthikul, *Ind. Eng. Chem. Res.* **48**, 2717-2720 (2009).
13. T. C. Drage, A. Arenillas, K. M. Smith, C. E. Snape, *Microporous Mesoporous Mater.* **116**, 504-512 (2008).
14. M. L. Pinto, L. Mafra, J. M. Guil, J. Pires, J. Rocha, *Chem. Mater.* **23**, 1387-1395 (2011).
15. J. P. Warren, J. D. Roberts, *J. Phys. Chem.* **78**, 2507-2511 (1974).
16. J. M. Burns, M. E. Ashley, G. C. Crockett, T. H. Koch, *J. Am. Chem. Soc.* **99**, 6924-6928 (1977).
17. H. R. Kricheldorf, *Org. Magn. Reson.* **14**, 198-203 (1980).
18. W. McGhee, D. Riley, K. Christ, Y. Pan, B. Parnas, *J. Org. Chem.* **60**, 2820-2830 (1995).
19. I. George, P. Viel, C. Bureau, J. Suski, G. Lecayon, *Surf. Interf. Anal.* **24**, 774-780 (1996).
20. B. Luo, J. E. Rossini, W. L. Gladfelter, *Langmuir* **25**, 13133-13141 (2009).
